# Supplementary material for: A Humanized Yeast Phenomic Model of Deoxycytidine Kinase to Predict Genetic Buffering of Nucleoside Analog Cytotoxicity
Source: Genes (Basel). 2019 Sep 30;10(10):770. doi: 10.3390/genes10100770 (PMC6826991; doi:10.3390/genes10100770)

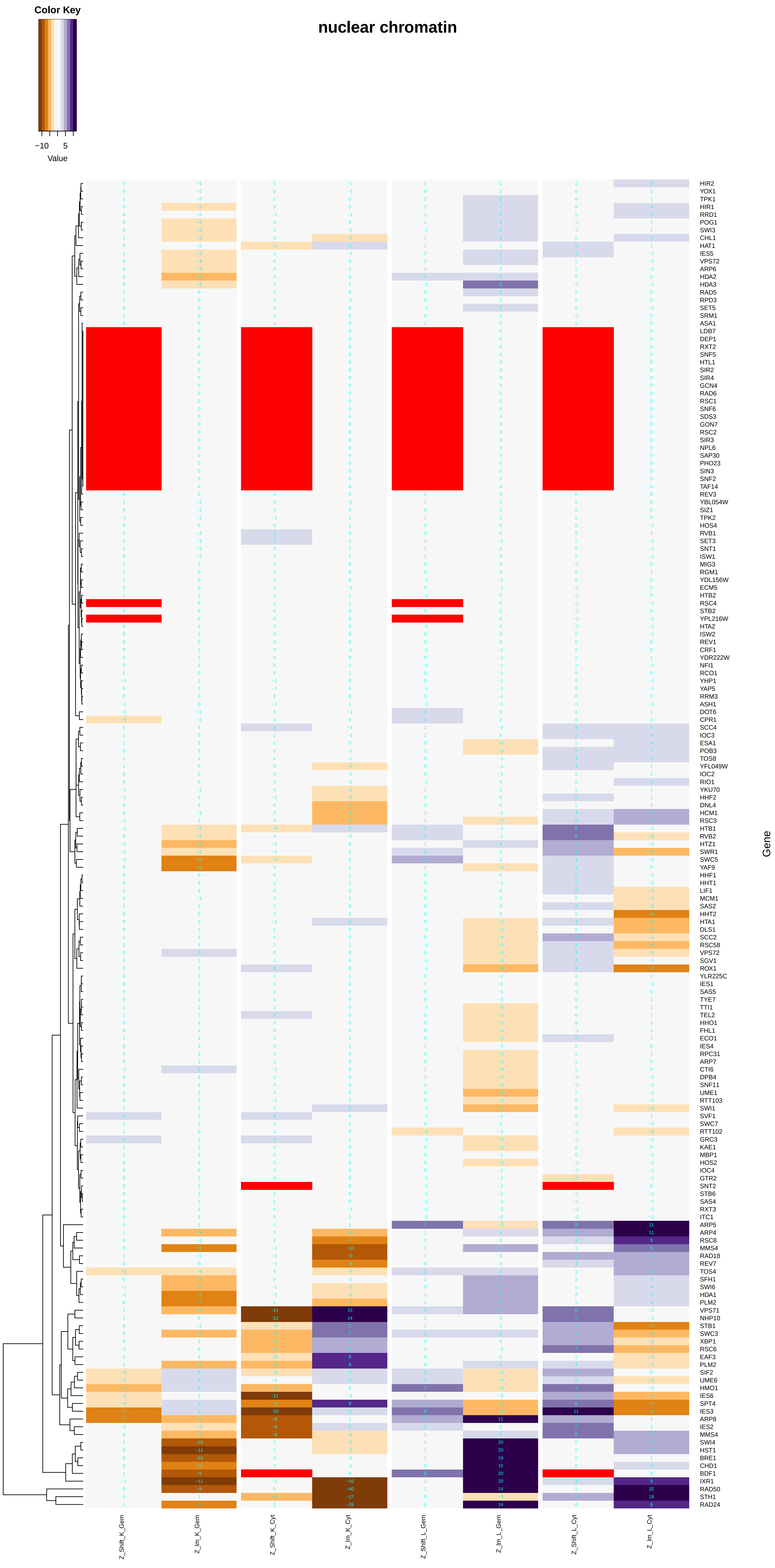

Color Key
−10
5
Value
nuclear chromatin
HIR2
YOX1
TPK1
HIR1
RRD1
POG1
SWI3
CHL1
HAT1
IES5
VPS72
ARP6
HDA2
HDA3
RAD5
RPD3
SET5
SRM1
ASA1
LDB7
DEP1
RXT2
SNF5
HTL1
SIR2
SIR4
GCN4
RAD6
RSC1
SNF6
SDS3
GON7
RSC2
SIR3
NPL6
SAP30
PHO23
SIN3
SNF2
TAF14
REV3
YBL054W
SIZ1
TPK2
HOS4
RVB1
SET3
SNT1
ISW1
MIG3
RGM1
YDL156W
ECM5
HTB2
RSC4
STB2
YPL216W
HTA2
ISW2
REV1
CRF1
YDR222W
NFI1
RCO1
YHP1
YAP5
RRM3
ASH1
DOT6
CPR1
SCC4
IOC3
ESA1
POB3
TOS8
YFL049W
IOC2
RIO1
YKU70
HHF2
DNL4
HCM1
RSC3
HTB1
RVB2
HTZ1
SWR1
SWC5
YAF9
HHF1
HHT1
LIF1
MCM1
SAS2
HHT2
HTA1
DLS1
SCC2
RSC58
VPS72
SGV1
ROX1
YLR225C
IES1
SAS5
TYE7
TTI1
TEL2
HHO1
FHL1
ECO1
IES4
RPC31
ARP7
CTI6
DPB4
SNF11
UME1
RTT103
SWI1
SVF1
SWC7
RTT102
GRC3
KAE1
MBP1
HOS2
IOC4
GTR2
SNT2
STB6
SAS4
RXT3
ITC1
ARP5
ARP4
RSC8
MMS4
RAD18
REV7
TOS4
SFH1
SWI6
HDA1
PLM2
VPS71
NHP10
STB1
SWC3
XBP1
RSC6
EAF3
PLM2
SIF2
UME6
HMO1
IES6
SPT4
IES3
ARP8
IES2
MMS4
SWI4
HST1
BRE1
CHD1
BDF1
IXR1
RAD50
STH1
RAD24
Gene
Z_Shift_K_Gem
Z_Im_K_Gem
Z_Shift_K_Cyt
Z_Im_K_Cyt
Z_Shift_L_Gem
Z_Im_L_Gem
Z_Shift_L_Cyt
Z_Im_L_Cyt

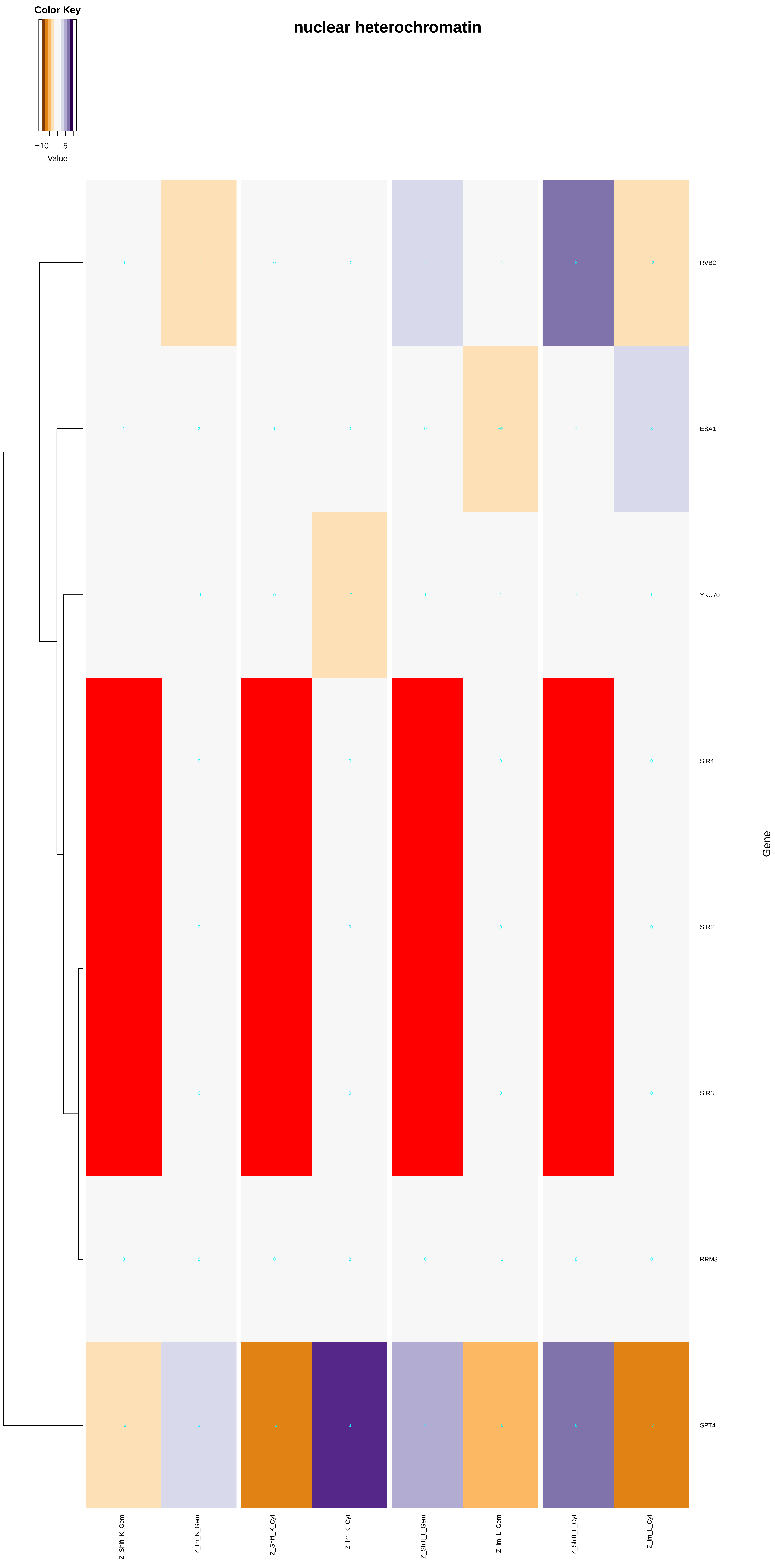

nuclear heterochromatin
Color Key
−10
5
Value
RVB2
ESA1
YKU70
SIR4
SIR2
SIR3
RRM3
SPT4
Gene
Z_Shift_K_Gem
Z_Im_K_Gem
Z_Shift_K_Cyt
Z_Im_K_Cyt
Z_Shift_L_Gem
Z_Im_L_Gem
Z_Shift_L_Cyt
Z_Im_L_Cyt

ASTRA complex

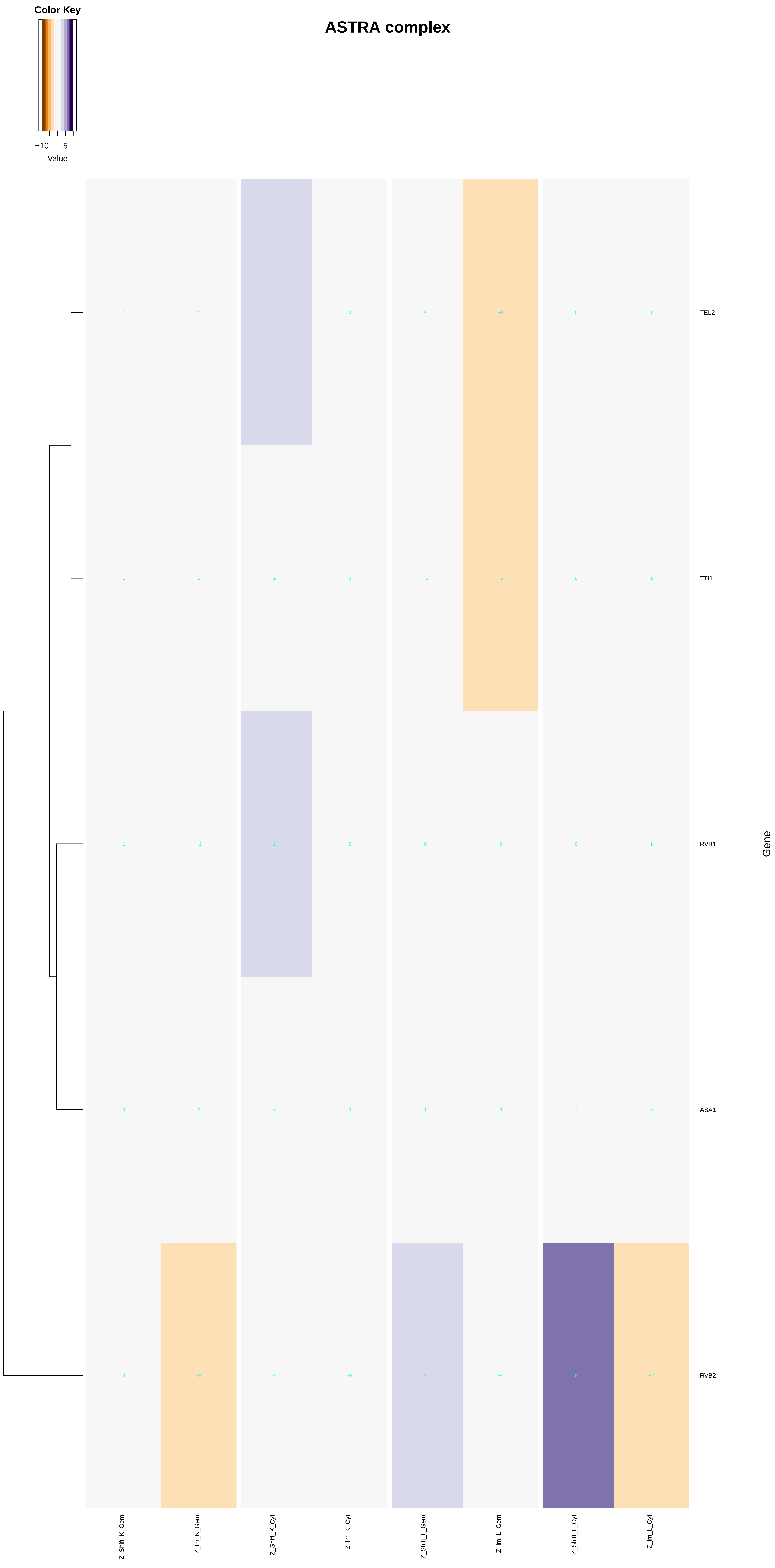

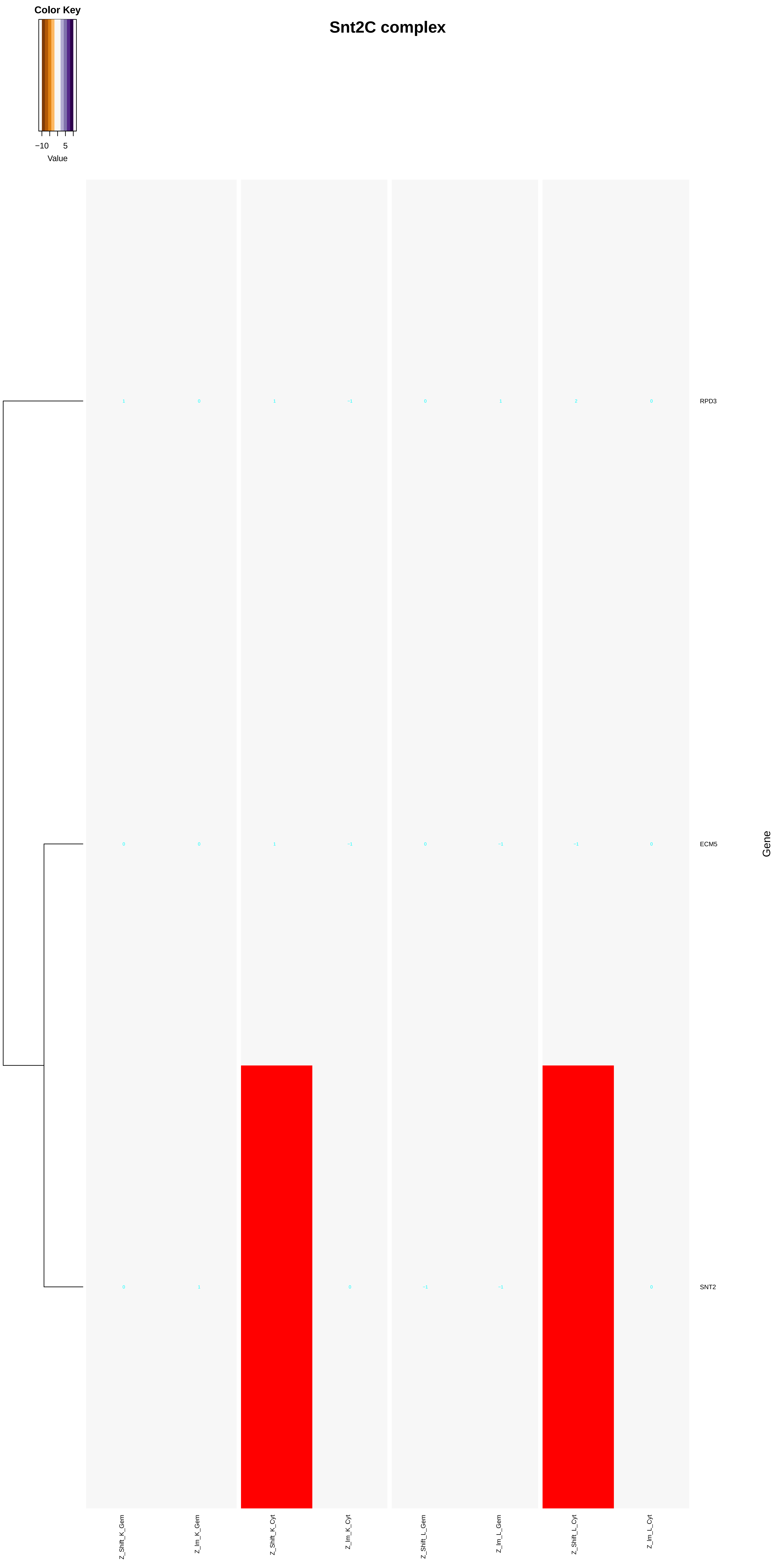

Color Key
−10
5
Value
Snt2C complex
RPD3
ECM5
SNT2
Gene
Z_Shift_K_Gem
Z_Im_K_Gem
Z_Shift_K_Cyt
Z_Im_K_Cyt
Z_Shift_L_Gem
Z_Im_L_Gem
Z_Shift_L_Cyt
Z_Im_L_Cyt

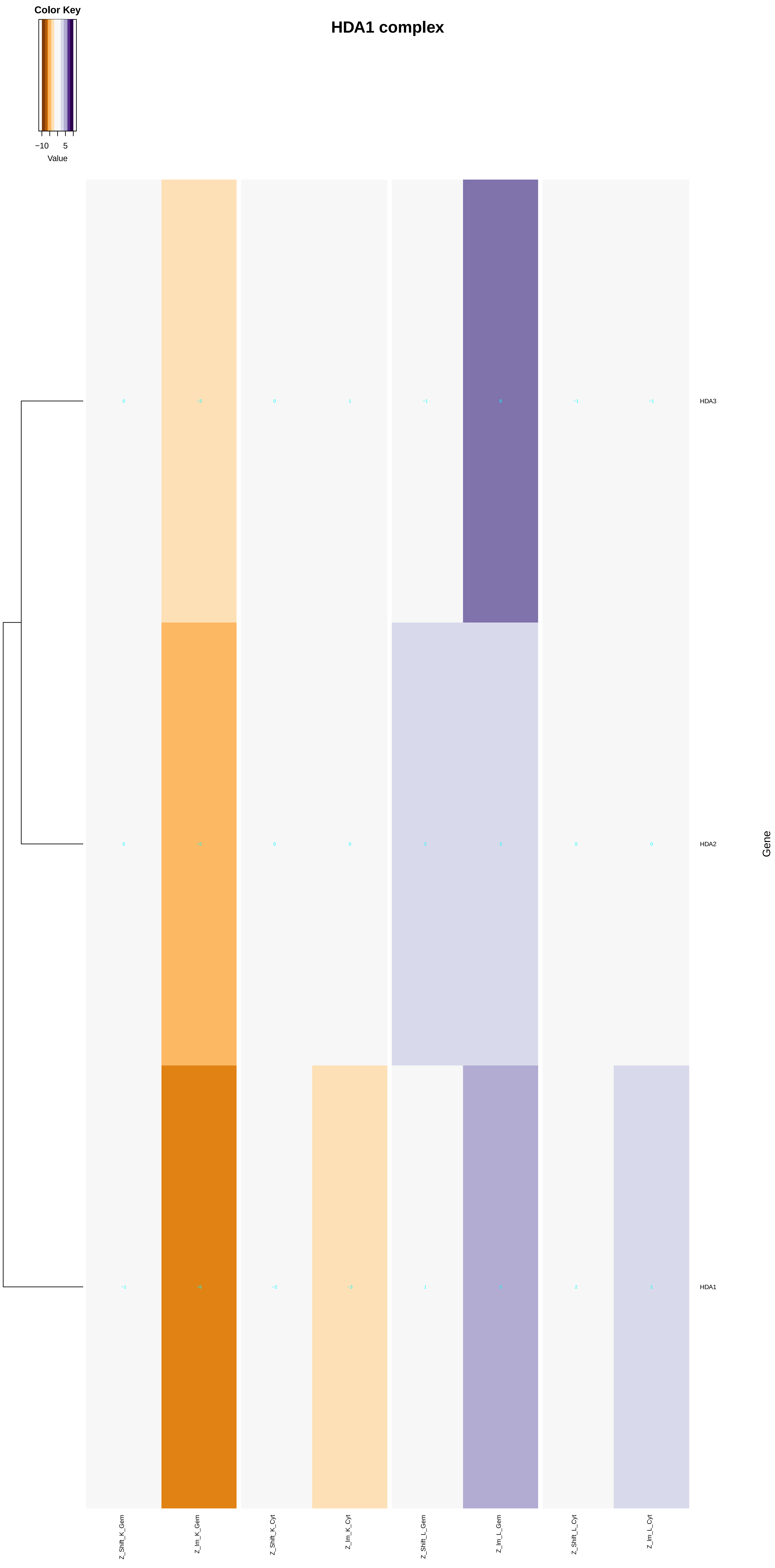

HDA1 complex
Color Key
−10
5
Value
HDA3
HDA2
HDA1
Gene
Z_Shift_K_Gem
Z_Im_K_Gem
Z_Shift_K_Cyt
Z_Im_K_Cyt
Z_Shift_L_Gem
Z_Im_L_Gem
Z_Shift_L_Cyt
Z_Im_L_Cyt

nuclear telomeric heterochromatin

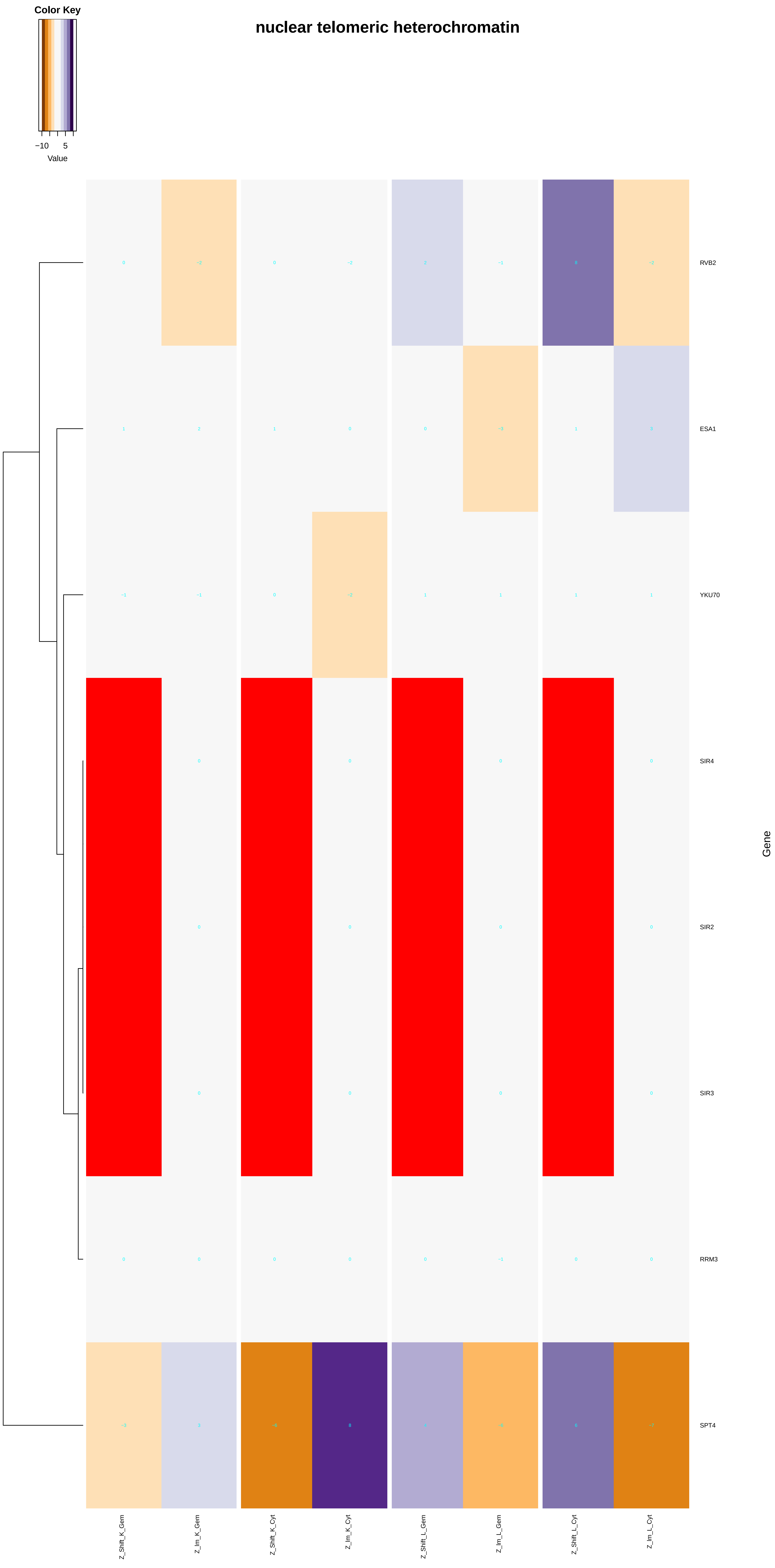

Supplement: Supplementary file 1 [file genes-10-00770-s001.zip › Additional_Files/Additional_File7_GOTermHeatmaps.zip!/Additional_File7_GOTermHeatmaps/nuclear_chromatin.pdf]
